# Wheat Quality Formation and Its Regulatory Mechanism

**DOI:** 10.3389/fpls.2022.834654

**Published:** 2022-03-30

**Authors:** Yanchun Peng, Yun Zhao, Zitong Yu, Jianbin Zeng, Dengan Xu, Jing Dong, Wujun Ma

**Affiliations:** ^1^Hubei Key Laboratory of Food Crop Germplasm and Genetic Improvement, Food Crops Institute, Hubei Academy of Agricultural Sciences, Wuhan, China; ^2^College of Agronomy, Qingdao Agricultural University, Qingdao, China; ^3^Food Futures Institute and College of Science, Health, Engineering and Education, Murdoch University, Perth, WA, Australia

**Keywords:** wheat quality, fertilization, watering regime, regulatory mechanism, sulfur deficiency

## Abstract

Elucidation of the composition, functional characteristics, and formation mechanism of wheat quality is critical for the sustainable development of wheat industry. It is well documented that wheat processing quality is largely determined by its seed storage proteins including glutenins and gliadins, which confer wheat dough with unique rheological properties, making it possible to produce a series of foods for human consumption. The proportion of different gluten components has become an important target for wheat quality improvement. In many cases, the processing quality of wheat is closely associated with the nutritional value and healthy effect of the end-products. The components of wheat seed storage proteins can greatly influence wheat quality and some can even cause intestinal inflammatory diseases or allergy in humans. Genetic and environmental factors have great impacts on seed storage protein synthesis and accumulation, and fertilization and irrigation strategies also greatly affect the seed storage protein content and composition, which together determine the final end-use quality of wheat. This review summarizes the recent progress in research on the composition, function, biosynthesis, and regulatory mechanism of wheat storage proteins and their impacts on wheat end-product quality.

## Introduction

Wheat (*Triticum aestivum*) is one of the largest grain crops in the world, and its quality mainly comprises processing and nutritional quality. The term “wheat quality” usually refers to the processing quality, which is mainly dependent on the content and characteristics of storage proteins in wheat grains (Shewry and Halford, 2002; Ma et al., 2019) and directly determines wheat’s market price and end-use value. Since storage proteins contain some components that can cause human intestinal inflammatory diseases or allergy, the concept of wheat “protein quality” is often used to cover the scope of the processing and nutritional quality (Scherf et al., 2016a).

Wheat processing quality is represented by the physical and chemical characteristics of the dough, which make it possible to process wheat into a variety of food products (Payne, 1987; He et al., 2005; Don et al., 2006; Zhang et al., 2007, 2014b, 2021; Li et al., 2015; Gao et al., 2016b; Chen et al., 2019; Jiang et al., 2019). Dough properties are mainly determined by gluten proteins, glutenins, and gliadins (Shewry and Halford, 2002). Glutenins can be subdivided into high molecular weight glutenin (HMW-GS) and low molecular weight glutenin (LMW-GS; Shewry et al., 2002; Veraverbeke and Delcour, 2002). HMW-GS is the main factor determining gluten elasticity, which is encoded by the *Glu-1* genes including *Glu-A1*, *Glu-B1,* and *Glu-D1* loci on the long arm of chromosomes 1A, 1B, and 1D, respectively. Each locus comprises two linked genes encoding two different types (X type and Y type) of HMW-GS subunits (McIntosh et al., 1991; Liu et al., 2003; Sun et al., 2006; Zheng et al., 2011; Peng et al., 2015; Yu et al., 2019). Gliadins are mainly monomer proteins, including ω-, α/β-, and γ-gliadins (Kasarda et al., 1984; Zhou et al., 2022). According to the Chinese National Standard (Wang et al., 2013), wheat can be divided into four categories based on the usage and gluten strength: (1) Strong gluten wheat: the endosperm is hard and the wheat flour produces very strong gluten, which is suitable for baking bread; (2) Medium strong gluten wheat: the endosperm is hard and the gluten is rather strong and is suitable for making instant noodles, dumplings, steamed bread, noodles, and other foods; (3) Medium gluten wheat: the endosperm is hard and the gluten strength is moderate and is suitable for making noodles, dumplings, steamed bread, and other foods; and (4) Weak gluten wheat: the endosperm is soft and the gluten is weak and is suitable for making cake, biscuit, and other foods. Strong gluten dough has high ductility resistance and can maintain stability (Ma et al., 2019). The dough can retain the gas produced during fermentation in discrete cells evenly distributed in the dough (Don et al., 2006). A lower gluten strength can cause the excessive expansion of gas cells during baking, resulting in the collapse of cell walls and aggregation of cells, and thereby a rough bread texture (Don et al., 2006). Therefore, strong gluten wheat has always been an important goal of wheat breeding programs.

Generally, the protein content in wheat grains ranges from 10 to 18% (Qi et al., 2012; Liu et al., 2018). To some extent, the protein content is positively correlated with wheat processing quality, particularly dough strength. However, the protein content and grain yield are usually negatively correlated with each other (Kibite and Evans, 1984). In real production, a large amount of nitrogen fertilizer is often applied in order to promote wheat yield and protein content, which tends to reduce the nitrogen use efficiency and cause negative impacts on the environment (Justes et al., 1994). In recent years, multiple methods have been developed with the aim to simultaneously improve wheat yield and protein content, such as the utilization of new genes and optimization of water and fertilization regimes (Alhabbar et al., 2018; Balotf et al., 2018; Roy et al., 2018, 2020, 2021; Yang et al., 2018; Yu et al., 2018a,b; Li et al., 2021a). However, the effect of protein content on wheat quality is rather complex due to the presence of gliadin components in the storage protein. Gliadins have an odd number of cysteine residues and a negative effect on wheat processing quality (Lindsay and Skerritt, 1999; Wieser, 2007; Rasheed et al., 2014). Therefore, high-quality wheat should be characterized by a higher content of glutenins and a lower content of gliadins, and wheat processing quality is not necessarily related to the grain protein content.

In Australia, researchers have been pursuing the breeding goal of wheat varieties with low-protein content but high quality since 2000, targeting at the improvement of wheat quality by optimizing the gluten composition, namely, a higher glu/gli ratio (Roy et al., 2018, 2020, 2021). In this approach, the protein content is no longer a target. Since there is a negative correlation between the grain protein content and yield, a low-protein content naturally means a higher yield without sacrificing the quality. However, considering the nutritional value of protein, the breeding goal of low-protein and high-quality wheat is not suitable for some developing countries. Therefore, “three-high wheat” (high quality, high yield, and high protein) should be the breeding goal for most countries.

## Genetics and Applications in Relation to Wheat Quality Breeding

Wheat quality can be improved by manipulation of the main storage protein genes. As a matter of fact, many effective genes have been efficiently utilized for decades, such as *GluD1* (5 + 10) and *GluB1* (17 + 18; Payne et al., 1981, 1987; Payne, 1987; Altpeter et al., 2004; Mohan and Gupta, 2015). The common HMW-GS alleles have been assigned with quality scores to facilitate their application in breeding (Payne et al., 1987). Although there are six HMW-GS genes in the wheat genome, most hexaploid wheat varieties only have three to five HMW glutenin subunits due to the silencing of some genes (Ma et al., 2003), such as the genes encoding the Ay subunit (Yu et al., 2019). Roy et al. (2018, 2020, 2021) found that the expression of Ay subunit has positive effects on grain protein content, grain yield, and quality. A new storage protein family consisting of the avenin-like proteins has also been identified to have great breeding value for the improvement of wheat quality (Chen et al., 2016). Since the genetic control of wheat quality has been comprehensively reviewed (Shewry and Tatham, 1997; Vasila and Anderson, 1997; Gras et al., 2001; She et al., 2011; Ortolan and Steel, 2017; Ma et al., 2019; Sharma et al., 2020; Wang et al., 2020), this review will not focus on this aspect.

## Manipulation of Fertilization and Watering Regimes

**Figure 1:**
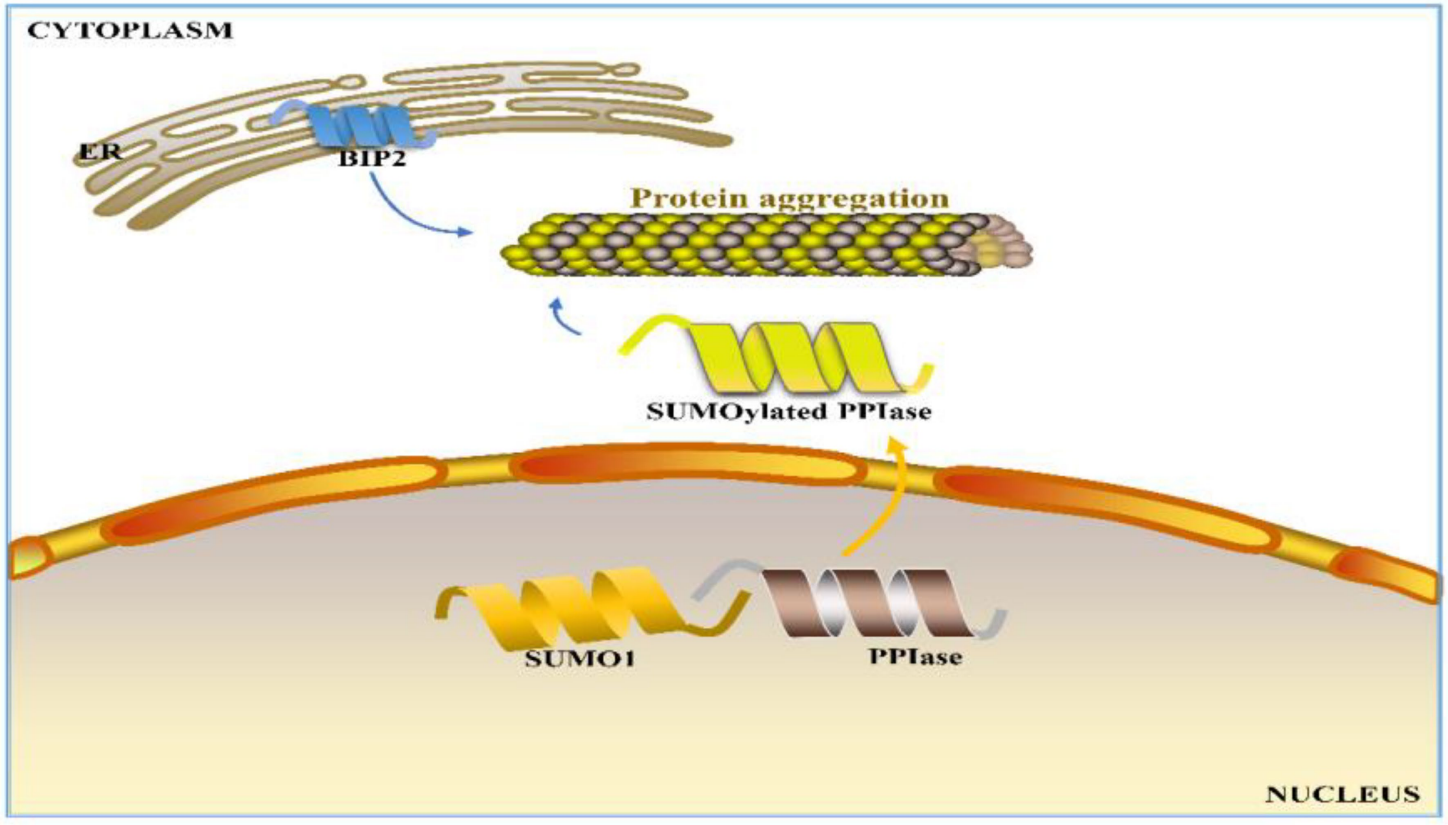
Proposed N-regulated mechanism for wheat grain protein polymerization in the cytoplasm. ER: endoplasmic reticulum, BIP2: luminal-binding protein 2 precursor, SUMO1: small ubiquitin-related modifier 1, and PPIase: peptidyl-prolyl cis-trans isomerase.

Seed storage proteins can account for 40–60% of wheat processing quality (Békés et al., 2006), and those unaccounted quality variations can be attributed to environmental factors. In wheat production, fertilization and watering strategies are also often considered for quality improvement (Li et al., 2018, 2019b; Yu et al., 2018a, 2021). As nitrogen (N) is one of the most important and essential elements for wheat, N fertilizer is usually the most efficient input for simultaneously increasing grain protein content and grain yield in wheat production (Zebarth et al., 2007; Malik et al., 2013; Zhen et al., 2018, 2020; Zhong et al., 2018, 2020; Ding et al., 2020; Xia et al., 2020; Hermans et al., 2021; Landolfi et al., 2021; Lyu et al., 2021; Dong et al., 2022; Liu et al., 2022; Ma et al., 2022). Kichey et al. (2007) demonstrated that 50–95% of nitrogen in mature grains is derived from the remobilization of nitrogen stored in the tissues before anthesis. However, nitrogen applied later in the growth period, namely, at anthesis or during grain filling, often increases grain protein content (Gooding and Davies, 1992; Sultana et al., 2017; Zhong et al., 2018, 2020). Li et al. (2018, 2019b) reported that nitrogen application during the grain filling period in winter wheat can significantly increase the uptake and accumulation of nitrogen. Yu et al. (2018a) reported that apart from the influence of genotype, grain yield and protein content have similar responses to nitrogen availability, with the former being slightly more sensitive than the latter. Furthermore, Yu et al. (2018a) proposed an N-mediated mechanism for protein polymerization in wheat grains: N promotes PPIase SUMOylation by interacting with SUMO1, facilitating the transport of PPIase into cytoplasm to support the formation of protein polymers (Yu et al., 2018a; Figure 1). Zhong et al. (2018, 2020) reported that at the same N application rate (240 kg ha^−1^), N topdressing can better promote the protein content and quality of wheat grains at the emergence of the top first leaf than at the emergence of the top third leaf of the main stem. The timing of N topdressing can significantly regulate γ-gliadins and HMW-GSs, while has almost no effect on the LMW-GS level, leading to a higher HMW-GS/LMW-GS ratio (Zhong et al., 2018). Furthermore, a delay of N topdressing was found to alter the grain hardness and flour allergenicity (Zhong et al., 2019). Ding et al. (2020) found that an increase in total N provision (210–270 kg ha^−1^) in the Yangtze River basin of China could enhance wheat grain yield, grain protein content, and nitrogen efficiency, with the appropriate topdressing timing and N application dose depending on the environment. Moreover, the biotic and abiotic stresses during wheat growth also significantly affect the quality of wheat (Duan et al., 2020). Among various stresses, drought has been identified to have a severe negative impact on wheat quality, particularly at the early grain filling stage (Gu et al., 2015). Usually, drought can cause stomatal closure, inhibit photosynthesis, increase respiration, and ultimately reduce starch biosynthesis, thereby leading to low yield of plants (Deng et al., 2018; Zhu et al., 2020). However, on the other hand, drought can enhance the content of wheat storage proteins to contribute to improved baking quality (Dong et al., 2012; Gu et al., 2015; Zhou et al., 2018). Different watering conditions were found to result in significant differences in the phosphorylation level of corresponding phosphoproteins in wheat grains (Zhang et al., 2014a). The changes in protein and starch synthesis during drought may be ascribed to post-translational protein modifications such as phosphorylation (Zhang et al., 2014a; Xia et al., 2018).

To reduce the yield loss caused by drought, moderate to high amounts of nitrogen fertilizer is often applied during wheat growth. A recent study showed that high-nitrogen fertilization under drought can increase the enzymatic protein synthesis for nitrogen and carbohydrate metabolism (Duan et al., 2020). Liu et al. (2022) reported that high-nitrogen treatments under drought conditions can either independently or coordinately facilitate the accumulation of wheat storage protein and gluten macropolymer, as well as improve lipid accumulation and protein secondary structure. The content of random coils and β-sheets of gluten proteins was also increased (Liu et al., 2022). These changes can contribute to the improvement of baking quality. Moreover, irrigation strategies under drought conditions have great impacts on crop yield and quality (Flagella et al., 2010; Xu et al., 2018b; Jha et al., 2019; Li et al., 2019b, 2021a). Li et al. (2021a) proposed an irrigation method that integrates micro-sprinkling irrigation and fertilizer, which could synergistically improve the grain yield and protein content of winter wheat. Compared with conventional irrigation, this method can reduce the total amount of water use and provide water and nitrogen at later growth stages, making more water and nitrogen available to wheat plants after flowering, which can reduce the canopy temperature and significantly delay leaf senescence and finally enhance the grain yield and protein content simultaneously.

**Figure 2:**
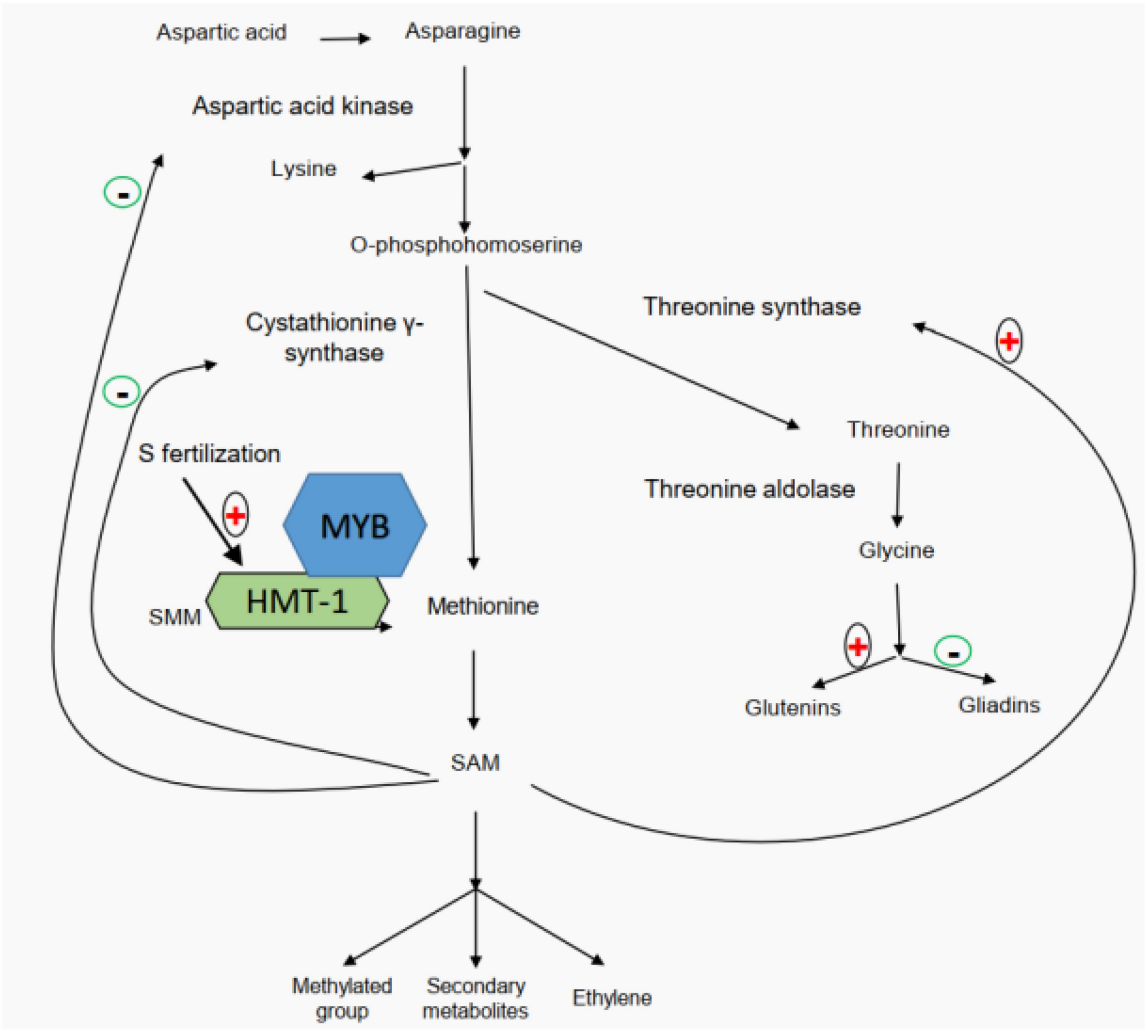
Sulfur-mediated regulation network of wheat gluten component biosynthesis (modified from Yu et al., 2021).

Also, studies of glutamine synthetase activity in wheat developing grains and flag leaves have demonstrated that high-nitrogen availability facilitates the participation of glutamine in biological processes (Yu et al., 2018a, 2021; Zhong et al., 2018, 2020). A number of studies have revealed that application of sulfur fertilizer can significantly improve wheat quality (Zhao et al., 1999a,b; Luo et al., 2000; Yu et al., 2021). Based on the differences in the distribution of cysteine residues among wheat gluten subunits, wheat storage proteins can be categorized into three types of subunits, including sulfur-poor subunits (ω-gliadins), sulfur-medium subunits (HMW-GS and α/β-gliadins), and sulfur-rich subunits (LMW-GS and γ-gliadins; Shewry et al., 1995). It is worth noting that this classification is based on the number of cysteine residues within each subunit instead of the total sulfur amount (Lindsay and Skerritt, 1999; Wieser, 2007; note: apart from cysteine, methionine is another sulfur containing amino acid). Since the disulfide bond is believed to be the foundation of gluten rheological properties, for a long time, it has been generally believed that sulfur’s positive effects on wheat quality are implemented through mediating the gluten component ratios based on their sulfur or cysteine contents (Ma et al., 2019). However, Yu et al. (2021) recently proposed a different regulatory mechanism through proteomics, transcriptomics, metabolomics, and field experiments (Figure 2). It clearly demonstrated that sulfur does not mediate the gluten component ratios based on their sulfur or cysteine contents (Yu et al., 2021). Their study showed that the application of sulfur enhances the accumulation of free glycine at the beginning of grain filling and then promotes the participation of glycine in glutenin biosynthesis. Glycine belongs to aspartate acid family, and its content disparity between gliadins (1.75%) and glutenins (13.33%) marks the main difference of the two gluten components (Yu et al., 2021). A higher content of free glycine under sulfur fertilizer treatment can more significantly promote the biosynthesis of glutenins than that of gliadins, resulting in a high glu/gli ratio (Yu et al., 2021). The gene network regulating the biosynthesis and accumulation of glutenin components is mediated by S-adenosylmethionine (SAM; Yu et al., 2021). In addition, a high concentration of SAM indicates that more secondary metabolites are involved in the final development of grains. Chen et al. (2014) found that the downregulation of SAM decarboxylase genes would reduce the rice grain length, pollen viability, seed setting rate, grain yield per plant, and abiotic stress (salinity, drought, and chilling) tolerance, indicating a positive effect of SAM on rice yield.

## Gene Networks Regulating Storage Protein Biosynthesis

The wheat storage protein genes have spatiotemporal specific expression, and generally function at the middle and late stage of seed development (Diaz et al., 2002; Dong et al., 2007; Gao et al., 2021). Although wheat storage protein synthesis is regulated by many factors, it is mainly regulated at the transcriptional level (Gao et al., 2021). In recent years, important progress has been achieved in research on the regulation of wheat storage protein synthesis (Table 1). A series of conserved *cis-*elements in the promoter region of wheat seed storage protein genes have been identified, including the bZIP binding sites (GCN4-like motifs, ATGAG/CTCAT and G-box motif, TTACGTGG), DNA binding with one finger (DOF) binding sites (PB-box, TGTAAAG), R2R3MYB-binding sites (AACAAC), RY repeat sites (RY-box, CATGCA), and other basal promoter elements (Aryan et al., 1991; Juhasz et al., 2011; Ravel et al., 2014; Guo et al., 2015; Makai et al., 2015). Thirty conserved motifs and three conserved *cis-*regulatory modules (CCRMs) were found within the 1-kb region upstream of the start codon of *Glu-1*: CCRM3 (−950 to −750), CCRM 2 (−650 to −400), and CCRM 1 (−300 to −101; Li et al., 2019a). All three CCRMs can regulate the expression of wheat storage proteins and the 300 bp promoter (−300 to −1) can ensure the precise initiation of *Glu-1* gene expression in the endosperm at 7 days after flowing and maintain its expression pattern during seed development. Further analysis revealed that CCRM1-1 (−208 to −101) is the core region for maintaining the endosperm-specific expression of *Glu-1* genes (Li et al., 2019a). In addition, various transcription factors (TFs) involved in gluten gene regulation have been identified, such as bZIP, DOF, MYB (myeloblastosis), and B3. A bZIP transcription factor SPA (storage protein activator) can bind to the GCN4-like motif (GLM and ATGAG/CTCAT) in the promoters of HMW-GS genes to enhance their expression in common wheat (Albani et al., 1997; Conlan et al., 1999; Ravel et al., 2014). Averagely, the expression intensity of SPA-B is 10- and 7-fold that of SPA-A and SPA-D, respectively (Ravel et al., 2009). SPA markers are associated with dough viscoelasticity such as dough strength, extensibility, and tenacity (Ravel et al., 2009). As a bZIP transcription factor, the SPA Heterodimerizing Protein (SHP) prevents the binding of SPA to the *cis-*motifs and represses the synthesis of both LMW-GS and HMW-GS (Boudet et al., 2019). Thus, the glu/gli ratio is decreased in common wheat (Boudet et al., 2019). Wheat prolamin-box binding factor (WPBF), a DOF transcription factor, was first identified from wheat as a homolog of barley prolamin-box binding factor (BPBF; Dong et al., 2007). WPBF binds the prolamin box of the gliadin promoter region and interacts with TaQM (cloned from the wheat root cDNA library; *QM*, initially found as a putative tumor suppressor gene) to activate the expression of LMW-GS and gliadin genes during wheat grain development (Dowdy et al., 1991; Mena et al., 1998; Dong et al., 2007). TaPBF-D, another DOF transcription factor, binds *in vitro* the prolamin box of *Glu-1By8* and *Glu-1Dx2* promoters and decreases their C-methylation level, and its overexpression was found to enhance HMW-GS accumulation in wheat grains (Zhu et al., 2018). TaGAMyb, a TF of the R2R3MYB family, binds to a C/TAACAAA/CC-like motif in the HMW-GS gene promoter, recruits the histone acetyltransferase TaGCN5, and activates the expression of the *Glu-1Dy* by facilitating the acetylation of histones H3K9 and H3K14 (Diaz et al., 2002; Guo et al., 2015). TaFUSCA3 is a wheat B3-superfamily TF specifically binding to the RY motif of the *Glu-1Bx7* promoter region to activate the *Glu-1Bx7* expression (Sun et al., 2017). TF interactions between TaSPA and TaFUSCA3 were discovered (Sun et al., 2017). It is well known that NAM-ATAF-CUC (NAC) TFs participate in a series of biological processes, including abiotic and biotic stress responses and organ development (Uauy et al., 2006; Xue et al., 2011; Liang et al., 2014; Borrill et al., 2017; Guerin et al., 2019). Recently, some NAC TFs (TaNAC019, TaNAC100, and TaSPR) in wheat have been identified to regulate grain storage protein synthesis (Gao et al., 2021; Li et al., 2021b; Shen et al., 2021). TaNAC019, a wheat endosperm-specific NAC TF, binds to the motif ([AT]NNNNNN[ATC][CG]A[CA]GN[ACT]A) in the promoter region of *Glu-1* genes. In coordination with TaGAMyb, it directly activates the expression of HMW-GS genes and indirectly modulates that of TaSPA (Gao et al., 2021). In a wheat natural population, two allelic variations of TaNAC019-B, TaNAC019-BI, and TaNAC019-BII were identified (Gao et al., 2021). TaNAC019-BI can improve flour processing quality and is an important candidate gene for wheat quality improvement (Gao et al., 2021). However, two recent studies demonstrated that both TaNAC100 and TaSPR function as repressors of seed storage protein expression in common wheat, indicating that further research is needed for better utilization of such TFs in breeding (Li et al., 2021b; Shen et al., 2021). The TaDME (wheat DEMETER) gene encoding 5-methylcytosine DNA glycosylase on the long arm of group 5 chromosomes suppresses the LMW-GS and gliadin gene expression by activating the demethylation of their promoters in the endosperm (Wen et al., 2012). It is worth noting that these studies have been mainly focused on the molecular regulatory mechanism of HMW-GS, LMW-GS, gliadins, or the total seed storage protein, and future research should be targeted at the regulatory mechanism for each subtype of gluten components, including different LMW-GSs (i-, m-, s-,α-, ω-, and γ-types) and gliadin components (α/β-, ω-, and γ-gliadins), so as to fine-tune wheat processing quality and improve the quality of wheat products for human consumption (Rasheed et al., 2014; Ma et al., 2019).

**Table 1:** The identified transcription factors regulate seed storage protein synthesis in wheat.

Transcription factor	Function	Target gene	Cis motif	Sequence	Reference
SPA	Transcriptional activation	glutenin promoters	G-box; GLM	ATGAG/CTCAT; ACGTG	Albani et al., 1997; Ravel et al., 2014
SHP	Transcriptional repression	glutenin promoters	G-box; GLM	ATGAG/CTCAT; ACGTG	Boudet et al., 2019
WPBF	Transcriptional activation	gliadin gene promoters	P-box	TGTAAAG	Mena et al., 1998; Dong et al., 2007
TaPBF-D	Transcriptional activation	HMW-GS gene promoters	P-box	TGTAAAG	Zhu et al., 2018
TaGAMyb	Transcriptional activation	HMW-GS gene promoters	unnamed	C/TAACAAA/CC	Diaz et al., 2002; Guo et al., 2015
TaFUSCA3	Transcriptional activation	HMW-GS gene promoters	RY-box	CATGCA	Sun et al., 2017
TaNAC019	Transcriptional activation	glutenin promoters	unnamed	[AT]NNNNNN[ATC][CG]A[CA]GN[ACT]A	Gao et al., 2021
TaNAC100	Transcriptional repression	HMW-GS gene promoters	unnamed	CATGT	Li et al., 2021b
TaSPR	Transcriptional repression	SSP gene promoters	unnamed	CANNTG	Shen et al., 2021

## Health Effects of Wheat Grains and the Underlying Regulatory Mechanism

Gluten can cause human diseases related to digestion of wheat flour products, such as celiac disease, non-celiac gluten sensitivity, and gluten allergy (Scherf et al., 2016a). The intake of too much proline-rich gluten can reduce pepsin activity in the gastrointestinal tract, resulting in the accumulation of flour polypeptides rich in Pro and Gln in the small intestine (Scherf et al., 2016a). Previous studies have demonstrated that gliadins are the most toxic wheat protein components related to celiac disease, and glutenins are classified as non-toxic or weakly toxic (Barone and Zimmer, 2016; Scherf et al., 2016a,b). In order to reduce the toxicity of wheat gluten, a variety of flour treatment methods have been developed, including chemical, physical, and enzymatic methods (Buddrick et al., 2015; Scherf et al., 2018; Xue et al., 2019; Abedi and Pourmohammadi, 2021). In addition, some genetic methods have also been used to knock out or silence gliadin coding genes. Generally, RNAi can reduce the content of total gliadin in wheat gluten by 60–80% (Gil-Humanes et al., 2010). However, some negative effects on the processing quality were observed in RNAi wheat lines (Gil-Humanes et al., 2010, 2014). For instance, CRISPR-Cas9 editing was applied to silence the α-gliadin gene to reduce immune reactivity by 85%, but the treatment also greatly reduced the gluten content by 85% and led to an obvious decline in processing quality (Sánchez-León et al., 2018). At present, the greatest challenge is to find a technical solution to reduce wheat gliadin and increase gluten content with high yield and high total protein.

Yu et al. (2021) showed that sulfur treatment can reduce sulfur-poor ω-gliadins (the most abundant among all gliadin subtypes) by up to 31.4% in the total gluten, particularly the ω5-gliadin known to cause WDEIA (wheat-dependent exercise-induced anaphylaxis disease), which could be reduced by 83.9%. The α/β-gliadins, ω1,2-gliadins, and γ-gliadins, which are known to cause celiac disease, were also reduced by up to 25.9% under sulfur treatment. Carcinogen acrylamide is a processing contaminant usually formed from free asparagine and reducing sugars through the Maillard reaction (Mottram et al., 2002; Stadler et al., 2002; Zyzak et al., 2003). It has been discovered in a range of baked, fried, roasted, and toasted foods, including bread, pies, cakes, biscuits, batter, and breakfast cereals (Raffan and Halford, 2019). Since free asparagine is the major precursor for the formation of acrylamide during food processing especially high temperature baking, its accumulation mechanism in wheat grains has emerged as a hot research topic (Mottram et al., 2002; Stadler et al., 2002; Raffan et al., 2021). In living cells, aspartate is the substrate of asparagine, which is formed through enzymes that catalyze the ATP-dependent transfer of an amino group from glutamine (Gaufichon et al., 2010). Five asparagine synthetase genes have been found in the wheat genome, including TaASN1, TaASN2, TaASN3.1, TaASN3.2, and TaASN4 (Xu et al., 2018a; Raffan and Halford, 2021). Among these genes, TaASN2 is seed-specific with the highest expression in the embryo (Gao et al., 2016a; Curtis et al., 2019). It has been revealed that free asparagine is commonly present in wheat even under normal growth conditions (Curtis et al., 2018). Both environmental factors and agricultural practice can affect its accumulation (Zhong et al., 2018, 2020). In addition, adverse growing conditions such as sulfur deficiency and pathogen infection can increase asparagine concentration (Raffan and Halford, 2019). World Health Organization^1^ has stated that acrylamide in the diet has potential cancer-causing effects. The food industry is in demand of available raw materials with lower acrylamide-forming potential. So far, numerous studies have been carried out to reduce acrylamide in wheat products, mainly by reducing the free asparagine concentration in wheat grains. For example, Muttucumaru et al. (2006) reported that sulfur application can reduce the asparagine accumulation in mature wheat grains, making the wheat products healthier for human daily consumption. More recently, Raffan et al. (2021) successfully reduced the asparagine concentration through CRISPR-Cas9 approach to knock out the six alleles of TaASN2, a seed-specific asparagine synthetase gene in wheat.

## Conclusion

The formation mechanism of wheat processing quality has been extensively studied *via* a broad range of biological approaches. Sulfur deficiency in soil has been reported as a global issue, which has negative impacts on wheat quality. An adequate level of sulfur fertilization is highly recommended in modern wheat farming to gain high processing quality as well as desirable nutritional value and healthy effect of the wheat end-products. Nitrogen fertilization after flowering should be considered for better processing quality. In the predicted drought season, low-protein content wheat cultivars may be selected for cultivation so that the grain starch can be allocated with more biosynthesis capacity to reduce yield loss. Molecular biological research has been mostly focused on the regulatory mechanism of the biosynthesis of various gluten components, which has led to the discovery of some key TFs that influence the quality. In future, TFs regulating specific HMW-GS subunits, LMW-GS types, and particularly the gliadin subtypes should be focused so that the relevant molecular markers can be used in breeding to meet a broad range of consumer needs.

## Author Contributions

YP, YZ, ZY, JZ, and DX prepared the first draft. JD and WM critically reviewed and revised the manuscript. All authors contributed to the article and approved the submitted version.

## Funding

This work is supported by National Natural Science Foundation of China (31701506), Youth Science Fund of Hubei Academy of Agricultural Sciences (2020NKYJJ03), and Fund of Hubei Key Laboratory of Food Crop Germplasm and Genetic Improvement (2020LZJJ04).

## Conflict of Interest

The authors declare that the research was conducted in the absence of any commercial or financial relationships that could be construed as a potential conflict of interest.

## Publisher’s Note

All claims expressed in this article are solely those of the authors and do not necessarily represent those of their affiliated organizations, or those of the publisher, the editors and the reviewers. Any product that may be evaluated in this article, or claim that may be made by its manufacturer, is not guaranteed or endorsed by the publisher.

## Footnotes

^1^ https://www.who.int/
